# Perforin Acts as an Immune Regulator to Prevent the Progression of NAFLD

**DOI:** 10.3389/fimmu.2020.00846

**Published:** 2020-05-22

**Authors:** Qian Wang, Dehai Li, Jing Zhu, Mingyue Zhang, Hua Zhang, Guangchao Cao, Leqing Zhu, Qiping Shi, Jianlei Hao, Qiong Wen, Zonghua Liu, Hengwen Yang, Zhinan Yin

**Affiliations:** ^1^Zhuhai Precision Medical Center, Zhuhai People's Hospital (Zhuhai Hospital Affiliated with Jinan University), Jinan University, Zhuhai, China; ^2^The Biomedical Translational Research Institute, Faculty of Medical Science, Jinan University, Guangzhou, China; ^3^The First Affiliated Hospital of Jinan University, Guangzhou, China

**Keywords:** perforin, NAFLD, CD4 T cells, IFN-γ, inflammation

## Abstract

Non-alcoholic fatty liver disease (NAFLD) is one of the main causes of cirrhosis and major risk factors for hepatocellular carcinoma and liver-related death. Despite substantial clinical and basic research, the pathogenesis of obesity-related NAFLD remains poorly understood. In this study, we show that perforin can act as an immune regulator to prevent the progression of NAFLD. Aged perforin-deficient (Prf^−/−^) mice have increased lipid accumulation in the liver compared to WT mice. With high-fat diet (HFD) challenge, Prf^−/−^ mice have increased liver weight, more severe liver damage, and increased liver inflammation when compared with WT controls. Mechanistic studies revealed that perforin specifically regulates intrinsic IFN-γ production in CD4 T cells, not CD8 T cells. We found that CD4 T cell depletion reduces liver injury and ameliorates the inflammation and metabolic morbidities in Prf^−/−^ mice. Furthermore, improved liver characteristics in HFD Prf^−/−^ and IFN-γR^−/−^ double knockout mice confirmed that IFN-γ is a key factor for mediating perforin regulation of NAFLD progression. Overall, our findings reveal the important regulatory role perforin plays in the progression of obesity-related NAFLD and highlight novel strategies for treating NAFLD.

## Introduction

Non-alcoholic fatty liver disease (NAFLD) pathogenesis is tightly linked to obesity and therefore is an emerging healthcare problem worldwide (1, 2). NAFLD, along with related inflammation, progressive subtype non-alcoholic steatohepatitis (NASH), fibrosis, and ultimately hepatocellular carcinoma, is becoming one of the leading causes of liver-related morbidity and mortality worldwide (3–5). The pathogenesis of NAFLD remains incompletely understood. It is appreciated that multiple concurrent intrahepatic and extrahepatic events contribute to development and progression of NAFLD, including cell senescence, insulin resistance, and immune system dysfunction (6, 7). Cellular senescence refers to the irreversible arrest of cell growth that occurs when cells are exposed to various stresses (8–10). Recent experimental evidence suggests that hepatocyte senescence is linked to the fibrosis that develops as NAFLD progresses; hepatocyte expression of p21, the universal cell cycle inhibitor, is positively correlated with fibrosis stage in liver sections from 70 NAFLD patients (11). Dysregulated lipid metabolism plays a key role in initiation and progression of hepatic steatosis and is frequently associated with inflammation of the liver (12, 13). Elevated inflammation promotes the development of insulin resistance, which in turn further promotes ectopic fat accumulation in the liver, thus forming a vicious cycle (14, 15). Inflammation and fibrogenesis are regulated by complex immunologic pathways that may present possible new therapeutic targets in the liver for NAFLD (7).

Perforin, which is primarily released by CD8^+^ T cells and natural killer (NK) cells, helps eliminate infected or dangerous cells and induce apoptosis (16, 17). Following degranulation, pores formed by perforin enable granzyme entry into cells and subsequent caspase activation. Perforin-mediated cytotoxicity is also involved in the homeostatic regulation of CD4 and CD8 T cells *in vivo* (18, 19). Recent reports revealed that perforin-mediated exocytosis (but not death-receptor-mediated apoptosis) is essential for immune surveillance of senescent cells, and disruption of this pathway as a result of disease or inflammation can lead to the accumulation of senescent cells in the liver (20). Interestingly, a recent study showed that mice on a high-fat diet (HFD) lacking perforin developed more severe obesity, glucose tolerance, and insulin resistance and had higher triglyceride levels in the liver when compared with wild-type (WT) controls (21). However, the precise role of perforin in the context of HFD-induced NAFLD has not been systematically researched yet.

We show that perforin acts as an important immune regulator to prevent NAFLD progression. Aged Prf^−/−^ mice had more severe liver injury and lipid accumulation than did WT control mice. In the condition of HFD-induced NAFLD, we also found that Prf^−/−^ mice developed more severe hepatic steatosis with more macrophage and IFN-γ, producing CD4^+^ T cell infiltration of the liver. Depletion of CD4^+^ T cells in Prf^−/−^ mice almost completely rescued the observed phenotypes, suggesting an important regulatory role for CD4^+^ T cells. Moreover, when IFN-γ receptor signaling is ablated by using perforin and IFN-γ receptor double knockout mice, both liver injury and lipid accumulation were dramatically diminished, indicating that IFN-γ signaling plays a pivotal role in mediating NAFLD pathogenesis.

Overall, our studies reveal that perforin acts as an important immune regulator for NAFLD progression. This finding expands our understanding of inflammation in regulating NAFLD and may have therapeutic implications for NAFLD in the future.

## Materials and Methods

### Mice

Prf^−/−^ and IFN-γR^−/−^ mice were purchased from the Jackson Laboratory. C57BL/6J mice were purchased from Guangdong Medical Laboratory Animal Center (Guangzhou, China). All mice were males and received either a normal control diet (SFD) or HFD (60 kcal % fat; Research Diets) beginning at an age of 6–8 weeks old. All mice were maintained under specified pathogen-free conditions at Jinan University (Guangzhou, China). Animal procedures were approved by and performed in accordance with the Jinan University's Institutional Laboratory Animal Care and Use Committee guidelines.

### Isolation of Liver Mononuclear Cells

The protocol used for isolating murine liver mononuclear cells (MNCs) was as described previously (22). Liver tissue was obtained from mice, and the tissue was dissociated to procure MNCs. To obtain liver MNCs, murine livers were pressed through a 200-gauge stainless steel mesh and suspended in either RPMI-1640 medium or PBS. The cells were then centrifuged at 50 g for 1 min. The cell suspension was collected and centrifuged again at 974 g for 10 min. The cell pellet containing MNCs was then resuspended in 40% Percoll (GE Healthcare, Uppsala, Sweden), after which the cell suspension was overlaid on 70% Percoll and centrifuged at 1,260 g for 30 min. The resulting cell pellets were collected from the interphase following two additional washings in PBS or RPMI-1640 medium.

### Serum Biochemistry

Mice were fasted overnight. Then, whole blood was collected, and serum alanine aminotransferase (ALT) and cholesterol levels were determined using an automatic biochemistry analyzer (7600-020, Hitachi, Japan).

### Cytokine Detection With ELISA

Mice were fasted overnight, and 0.1 g of liver tissue was harvested from the mice in 1 ml of PBS. Liver tissue was then homogenized by hand and centrifuged at 3,000 rpm for 10 min, after which the supernatant was carefully collected. All steps were performed at 4°C. IL-6, IFN-γ, and TNF-α levels in liver supernatants were determined using a commercially available mouse enzyme-linked immunosorbent assay (ELISA) kit (eBioscience, San Diego, CA, USA) according to the manufacturer's instructions.

### Flow Cytometry Analysis

Non-parenchymal cells were transferred to a new well and treated with 1:1000 GolgiPlug, 1 ng/ml ionomycin, and 50 ng/ml PMA for 4–6 h. Intracellular and cell surface staining was performed as described in the fixation/permeabilization kit (554714; BD) protocol. Cells were stained with the surface markers PEcy7-anti-mouse CD3, PE-anti-mouse NK1.1, FITC-anti-mouse CD4, and PerCPCY5.5-anti-mouse CD8 for 15 min at 4°C. Cells were stained for cytokines with BV421 anti–mouse IFN-γ and APC-IL-17A for 30 min at 4°C, washed with PBS, and analyzed using FACS verse flow cytometry (BD). Data were analyzed using FlowJo (TreeStar).

### CD4^+^ T Cell Depletion

To deplete CD4^+^ T cells, 200-μg doses of anti-CD4 monoclonal antibody (clone: GK1.5; Sungene Biotech) per mouse were intraperitoneally injected weekly during HFD challenge. Sterile-filtered PBS was used as a control.

### Histological Examination

Liver tissue was harvested and fixed in 4% (w/v) paraformaldehyde, and 4 mm-thick sections that had been affinized and rehydrated were stained with hematoxylin and eosin (H&E). Hepatic lipid content was determined using frozen sections embedded in Tissue-Tek O.C.T. compound and stained with Oil Red O (Sigma-Aldrich, St. Louis, MO, USA). Images were acquired on a Leica DM3000 microscope.

### Immunofluorescence

Liver tissue was harvested, fixed in 4% (w/v) paraformaldehyde, and cut in 4 mm-thick sections. Liver sections were then perfused with 30 ml of 4% paraformaldehyde for fixation. Sections were then incubated with the following dilutions of mouse-specific primary antibodies: 1:200 anti-F4/80 (ab16911, Abcam) and 1:200 iNOS antibody (GTX74171, Gentex). For visualization, 1:200 fluorescent Alexa Fluor 594 and FITC 488 secondary antibodies (Invitrogen Vector) were used for both individual staining and co-staining at room temperature for 2 h. After washing, tissue sections were fixed with Vectashield containing DAPI for visualization. A laser cofocal microscopy (TCS SP8, Leica) was used to capture images and conduct further analysis. For the microscopy images displaying M1 (iNOS+ F4/80+) or total macrophages (F4/80+), 4 slides per mouse liver tissue were prepared and 4 fields were captured from each slide. The quantification of M1 or total macrophages was conducted in these 16 fields and designated as one biological independent sample, and the percentage of M1 in total macrophages was calculated and shown.

### Tissue Triglyceride Quantification

The protocol for quantifying hepatic triglyceride (TG) levels was carried out as described previously (23). Briefly, 20–30 mg of liver tissue was homogenized in 500 μl of PBS and mixed with chloroform/methanol 2:1 (vol/vol). The organic phase was transferred, air-dried overnight, and resuspended in 1% Triton X-100 in absolute ethanol. The concentration of TGs was then quantified using a serum triglyceride determination kit (Sigma, Triglyceride Reagent T2449 and Free Glycerol Reagent F6428).

### RNA Extraction and Quantitative Real-Time PCR

Total liver RNA was isolated using TRIzol Reagent (DP424, Tiangen, China). cDNA synthesis was performed using a Prime Script RT Reagent Kit (Takara, Shiga, Japan). Levels of mRNA expression were quantified by real-time PCR (RT-PCR). RT-PCR was performed using TB Green (Takara). Primer sequences are shown in the Table 1.

**Table 1:** Primers for real-time RT-PCR.

Hprt forward	5^′^-CGTCGTGATTAGCGATGATGAAC-3^′^
Hprt reverse	5^′^-TCACTAATGACACAAACGTGATTC-3^′^
Fabp4 forward	5^′^-GACGACAGGAAGGTGAAGAG-3^′^
Fabp4 reverse	5^′^-ACATTCCACCACCAGCTTGT-3^′^
Cebpα forward	5^′^-AAGAACAGCAACGAGTACCGG-3^′^
Cebpα reverse	5^′^-CATTGTCACTGGTCAGCTCCA-3^′^
SREBP-1C forward	5^′^-GATCAAAGAGGAGCCAGTGC-3^′^
SREBP-1C reverse	5^′^-TAGATGGTGGCTGCTGAGTG-3^′^
PPARγ forward	5^′^-GCCCTTTGGTGACTTTATGG-3^′^
PPARγ reverse	5^′^-CAGCAGGTTGTCTTGGATGT-3^′^
PPARα forward	5^′^-TCGGACTCGGTCTTCTTGAT-3^′^
PPARα reverse	5^′^-TCTTCCCAAAGCTCCTTCAA-3^′^
Cox-1 forward	5^′^-CTCACAGTGCGGTCCAAC-3^′^
Cox-1 reverse	5^′^-CCAGCACCTGGTACTTAA-3^′^
AOX forward	5^′^-TCGGGCAAGTGAGGCGCATT-3^′^
AOX reverse	5^′^-AGCAACAGCATTGGGGCGGA-3^′^
Cpt1α forward	5^′^-CCCAAGTATCCACAGGGTCA-3^′^
Cpt1α reverse	5^′^-TTTGAATCGGCTCCTAATGG-3^′^
Lipe forward	5^′^-GTGGAGGCACATTTAGTTCT-3^′^
Lipe reverse	5^′^-GTGACCTGTTTGTTTGTTCT-3^′^
Lpl forward	5-TAGATGAGGCCAACCTGTCC-3^′^
Lpl reverse	5-CTGCGTAGTCGGGGTACATT-3^′^
CD36 forward	5^′^-AGATGACGTGGCAAAGAACAG-3^′^
CD36 reverse	5^′^-CCTTGGCTAGATAACGAACTCTG-3^′^
Scd1 forward	5^′^-TTCTTGCGATACACTCTGGTGC-3^′^
Scd1 reverse	5^′^-CGGGATTGAATGTTCTTGTCGT-3^′^
Cidea forward	5^′^-TGACATTCATGGGATTGCAGAC-3^′^
Cidea reverse	5^′^-GGCCAGTTGTGATGACTAAGAC-3^′^
Chrebpβ forward	5^′^-TCTGCAGATCGCGTGGAG-3^′^
Chrebpβ reverse	5^′^-CTTGTCCCGGCATAGCAAC-3^′^
Fasn forward	5^′^-CCTTGGCTAGATAACGAACTCTG−3^′^
Fasn reverse	5^′^-ATCCATAGAGCCCAGCCTTCCATC−3^′^

### Statistical Analysis

Data are presented as the mean ± SEM. Statistical significance between two groups was evaluated using a two-tailed unpaired Student's *t*-test. Values of *P* < 0.05 were considered to be statistically significant. The data shown in each panel of these figures were collected from a single experiment; each experiment was repeated for at least three times and showed consistent results. Moreover, the statistical analysis was conducted on each single experiment.

## Results

### Perforin Deficiency Accelerates Liver Injury and Enhances Lipid Accumulation in 14 Month-Old Mice

**Figure 1:**
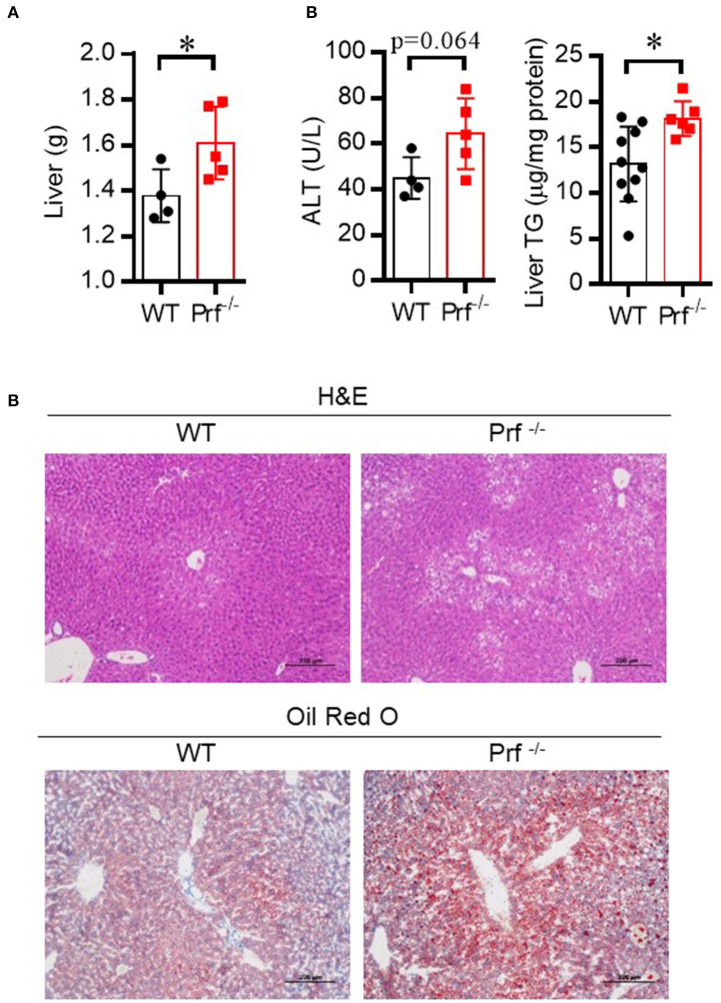
Perforin deficiency accelerates liver injury and enhances lipid accumulation in 14 month-old mice. **(A)** Liver weight was determined for WT and Prf^−/−^ mice on a normal chow diet (*n* = 4–5). **(B)** Serum levels of ALT (left, *n* = 4–5) and liver TG content (right, *n* = 6–10) were measured. **(C)** Representative images of liver sections stained with H&E and Oil red O in WT and Prf^−/−^ mice at the age of 14 months. ALT, alanine aminotransferase; WT, wild-type; TG, triglyceride. The data shown in each panel of the figures were collected from a single experiment, and each experiment was repeated for at least three times and rendered consistent results. Means ± SEM, *p < 0.05.

NAFLD is common in the elderly, in whom it carries a more substantial burden of hepatic (non-alcoholic steatohepatitis, cirrhosis, and hepatocellular carcinoma) and extra-hepatic manifestations and complications (cardiovascular disease, extrahepatic neoplasms) than in younger age groups (24). Aged Prf^−/−^ mice have been reported to have accumulation of senescent cells and development of chronic systemic and local inflammation in the liver (25, 26). We hypothesized that aged Prf^−/−^ mice might also have more severe hepatic morbidities since inflammation correlates with liver dysfunction. To test this hypothesis, we first determined liver weights and liver injury (ALT) levels in aged WT and Prf^−/−^ mice at 14 months of age. As expected, the aged Prf^−/−^ mice showed significantly increased liver weight (Figure 1A), elevated liver damage, and increased lipid accumulation as shown by levels of ALT that trended as increased and significantly increased liver TG levels (Figure 1B). Furthermore, liver histological analysis revealed more severe hepatic steatosis and significantly increased accumulation of lipid in aged Prf^−/−^ mice compared with WT mice (Figure 1C). These results indicated that the perforin deficiency aggravates liver injury and steatosis in aged mice.

### Perforin Deficiency Aggravates HFD-Induced Liver Injury and Steatohepatitis

**Figure 2:**
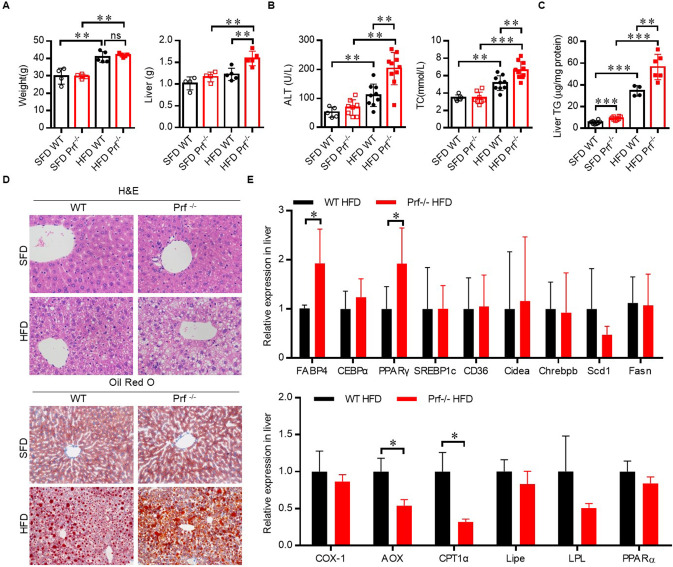
Perforin deficiency aggravates HFD-induced liver injury and steatohepatitis. WT and Prf^−/−^ mice were fed SFD or HFD for 10 weeks at 6–8 weeks of age. **(A)** Body weight (left panel) and liver weights (right panel) (*n* = 4–5), **(B)** measurements of serum ALT (left panel) and cholesterol levels (right panel) (*n* = 5–10), and **(C)** measurements of liver TG content (*n* = 4–8). **(D)** Representative images of liver sections stained with H&E and Oil red O. **(E)** Expression levels of lipogenic genes (top) and fatty acid oxidation genes (bottom) in the liver, relative to Hprt expression levels (*n* = 4–9). The data shown in each panel of the figures were collected from a single experiment, and each experiment was repeated for at least three times and rendered consistent results. Means ± SEM, *p < 0.05; **p < 0.01, ***p < 0.001.

The role of perforin in NAFLD was then investigated using an HFD-induced NAFLD model. Prf^−/−^ and WT mice at 6–8 weeks of age were fed on SFD or HFD for 10 weeks to induce NAFLD. As expected, HFD challenge was associated with elevated body weight and ALT activation in WT mice (Figures 2A,B). The increase in body weight in response to HFD challenge was comparable between WT and Prf^−/−^ mice; however, Prf^−/−^ mice had significantly enlarged livers (Figure 2A). Additionally, the Prf^−/−^ mice had more severe liver damage as indicated by higher ALT levels. The livers of HFD Prf^−/−^ mice exhibited significantly increased lipid accumulation (Figure 2C). Histological analysis of livers indicated that HFD Prf^−/−^ mice had more severe hepatic steatosis and lipid accumulation when compared with WT controls (Figure 2D). Moreover, RT-PCR analysis of liver samples from HFD mice showed that the expression levels of genes involved in lipid production such as fatty acid binding protein 4 (Fabp4) and peroxisome proliferator-activated receptor gamma (PPARγ) were significantly increased, whereas expression levels of lipid catabolism-related genes such as carnitine palmitoyl transferase 1 (CPT-1α) and aldehyde oxidase (AOX-1) were significantly decreased in HFD Prf^−/−^ mice (Figure 2E). These results indicated that perforin deficiency with HFD challenge aggravated liver injury and steatohepatitis.

### Perforin Deficiency Promotes an Inflammatory Response in the Liver After HFD Challenge

**Figure 3:**
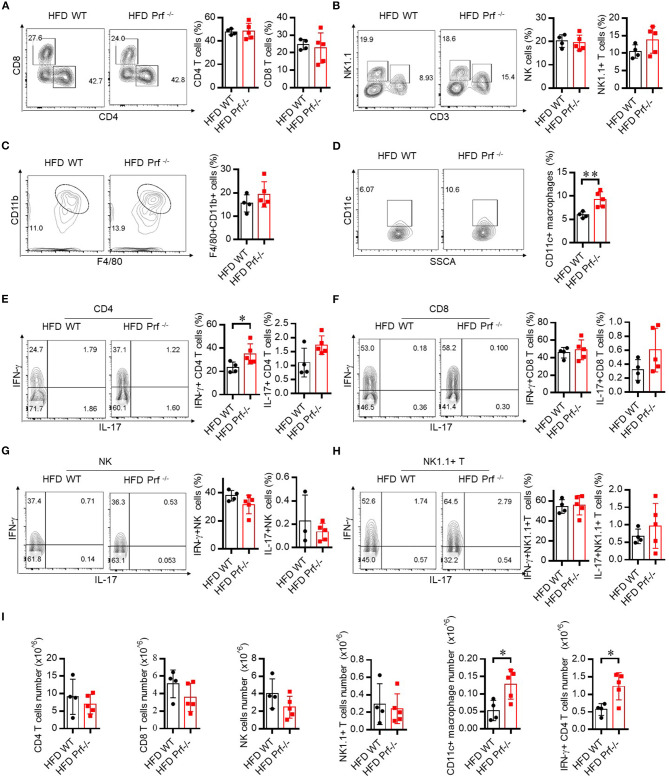
Perforin deficiency drives inflammatory reactions in liver after HFD challenge. WT and Prf^−/−^ mice were fed HFD for 10 weeks. Flow cytometry analysis of the representative histogram (left) and frequency (right) of liver CD4 T cells, CD8 T cells **(A)**, NK cells, NK1.1+ T cells **(B)**, macrophages **(C)**, and CD11c+ macrophages **(D)** and intracellular staining in the liver for IFN-γ and IL-17 in CD4 T cells **(E)**, CD8 T cells **(F)**, NK cells **(G)**, and NK1.1+ T cells **(H)**. **(I)** The total number of CD4 T cells, CD8 T cells, NK cells, and NK1.1+ T cells, CD11c+ macrophages and IFN-γ+CD4 T cells. *n* = 4–5 mice per group. The data shown in each panel of the figures were collected from a single experiment, and each experiment was repeated for at least three times and rendered consistent results. Means ± SEM; *p < 0.05.

Pro-inflammatory T cells promote M1 macrophage activation and intensively contribute to HFD-induced NAFLD (27). To explore the mechanism that drives more severe NAFLD in Prf^−/−^ mice, we analyzed the composition of the immune cell infiltrate in the liver by flow cytometry. Perforin deficiency did not alter the infiltration of CD4, CD8, NK, NK1.1+ T cells, or total macrophages in the liver (Figures 3A–C). However, the cell number of CD11c+ macrophages was significantly increased (Figure 3D). We next evaluated inflammatory cytokine production by these immune cell subsets. Interestingly, we observed that IFN-γ production from CD4 T, but not CD8 T cells, NK cells, or NK1.1+ T cells, was significant increased, while IL-17 was barely detectable and largely unaffected in all subsets (Figures 3E–H). We characterized the cell number in each category and found no significant difference of CD4 T cells, CD8 T cells, and NK and NK1.1+T cells. The cell numbers of CD11c+ macrophages (M1) and IFN-γ+CD4 T cells were significantly increased in perforin KO liver, which equivalent as the percentage analysis (Figure 3I).

**Figure 4:**
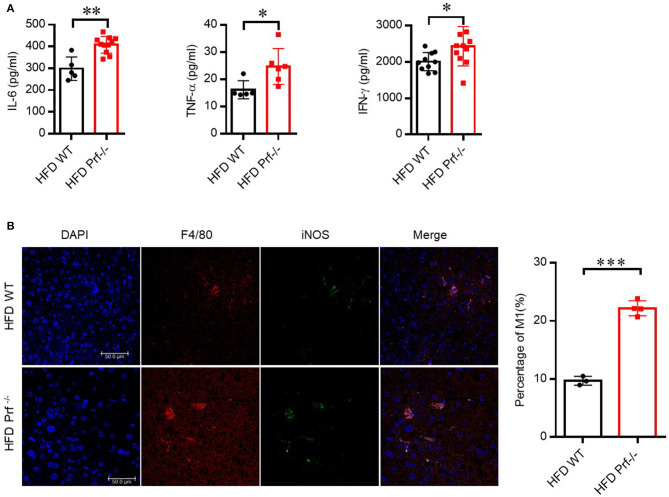
Perforin deficiency drives the accumulation of M1 macrophages and releases of pro-inflammatory cytokines in the liver with HFD challenge. WT and Prf^−/−^ mice were fed HFD for 10 weeks. **(A)** IL-6 (left panel, *n* = 5–11), TNF-α (middle panel, *n* = 5–6), and IFN-γ (right panel, *n* = 10–11) levels in liver supernatants. **(B)** Representative immunofluorescence images from liver with DAPI (blue), F4/80 (red), iNOS (green), and co-localization (merged image) (left) and the percentage of M1/M (refers to iNOS+F4/80+cells/F4/80+ cells) was calculated (right). The data shown in each panel of the figures were collected from a single experiment, and each experiment was repeated for at least three times and rendered consistent results. Means ± SEM, *p < 0.05; **p < 0.01; ***p < 0.001.

We also determined the levels of pro-inflammatory cytokines secreted by the liver in HFD-challenged Prf^−/−^ and WT mice. As expected, Prf^−/−^ livers produced more IL-6, TNF-α, and IFN-γ compared with livers from WT controls (Figure 4A). Immunofluorescence analysis showed that perforin deficiency robustly promoted the enrichment of M1 macrophage in the liver, which was consistent with the previous percentage and cell number analysis (Figure 4B). These findings suggest that upon HFD challenge, the IFN-γ level and M1 macrophage-mediated inflammation are enhanced in Prf^−/−^ mice.

### Perforin Regulates Fatty Liver Disease Through CD4 T Cells in the Liver

**Figure 5:**
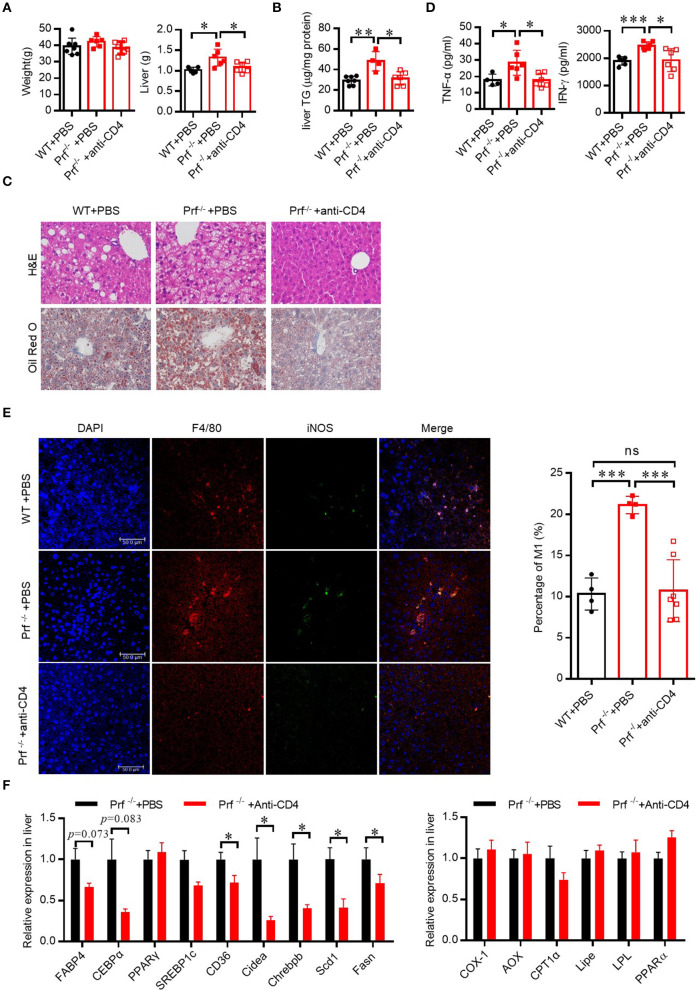
Perforin regulates fatty liver disease through CD4 T cells in the liver. WT and Prf^−/−^ mice were injected with PBS, or Prf^−/−^ mice were injected with an anti-CD4 antibody weekly during 10 weeks of HFD challenge**. (A)** Body and liver weight were measured (*n* = 6–8). **(B)** Measurements of liver TG content (*n* = 4–7). **(C)** Representative images of liver sections stained with H&E (top) and Oil red O (bottom). **(D)** Serum TNF-α and IFN-γ levels were determined (*n* = 4–6). **(E)** Representative immunofluorescence images from liver with DAPI (blue), F4/80 (red), iNOS (green), and co-localization (merged image) (left) and the percentage of M1/M (refers to iNOS+F4/80+cells/F4/80+ cells) was calculated (right). **(F)** Expression levels of lipogenic genes (left) and fatty acid oxidation genes (right) in the liver relative to Hprt expression levels (*n* = 6–11). The data shown in each panel of the figures were collected from a single experiment, and each experiment was repeated for at least three times and rendered consistent results. Means ± SEM, *p < 0.05; **p < 0.01, ***p < 0.001.

To determine whether increased IFN-γ production from CD4 T cells in Prf^−/−^ mice was associated with the exacerbated liver phenotypes that develop after HFD challenge, we depleted CD4 T cells in Prf^−/−^ mice and then fed the mice with HFD. As expected, CD4 T cell depletion predisposed Prf^−/−^ mice to decreased liver weights, lipid accumulation, and diminished liver damage (Figures 5A–C). Notably, levels of the pro-inflammatory cytokine TNF-α, as well as macrophage accumulation, were also significantly decreased following CD4 T cell depletion in Prf^−/−^ mice; so was the IFN-γ level, though CD4 T cells are not the only cells producing IFN-γ (Figures 5D,E). Furthermore, the mRNA expression levels of genes involved in lipogenesis such as Fabp4, CEBPα, PPARγ, SREBP1c, Chrebpβ, and Scd1 were decreased following CD4 T cell depletion in Prf^−/−^ mice, whereas lipolysis-related genes such as AOX1, CPT1α, and LPL were unchanged following CD4 T cell depletion in Prf^−/−^ mice (Figure 5F). These findings indicate that CD4 T cells play a critical role in perforin-mediated regulation of NAFLD progression.

### Hepatic Steatosis in Prf^-/-^ Mice Is Dependent on IFN-γ-Mediated Inflammation

**Figure 6:**
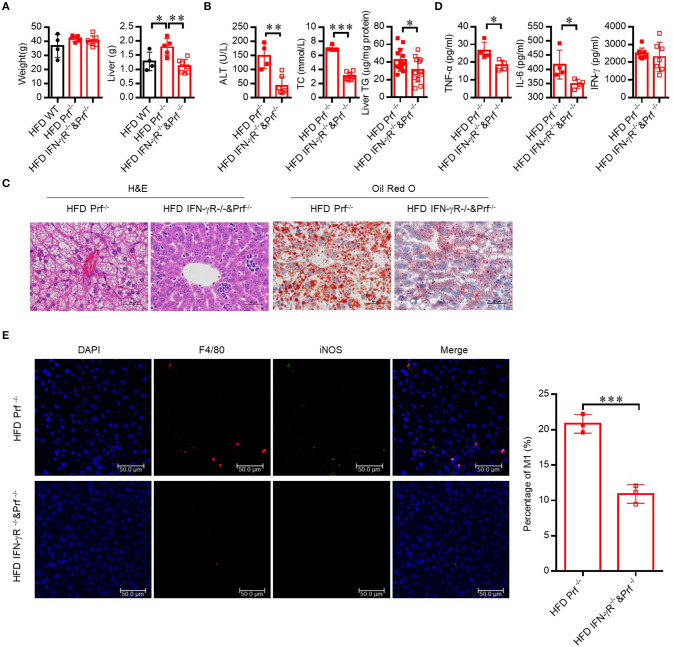
Hepatic steatosis in Prf^−/−^ mice is dependent on IFN-γ mediated inflammation. Prf^−/−^ and IFN-γR^−/−^ and Prf^−/−^ mice were fed HFD for 10 weeks. **(A)** Body and liver weight were measured (*n* = 4–7). **(B)** Serum levels of ALT (left panel, *n* = 4–7), cholesterol (middle panel, *n* = 4–7), and liver TG (right panel) were measured (*n* = 12–13). **(C)** Representative images of liver sections stained with H&E (left) and Oil red O (right). **(D)** TNF-α, IL-6, and IFN-γ levels were detected in liver culture supernatants (*n* = 4). **(E)** Representative immunohistochemistry of DAPI (blue), F4/80 (red), iNOS (green), and co-localization (merged image) in liver, and the percentage of M1/M (refers to iNOS+F4/80+cells/ F4/80+ cells) was calculated (right). The data shown in each panel of the figures were collected from a single experiment, and each experiment was repeated for at least three times and rendered consistent results. Means ± SEM, *p < 0.05; **p < 0.01, ***p < 0.001.

Since the level of IFN-γ was significantly increased in the livers of Prf^−/−^ mice, we next explored whether CD4 T cells contribute to exacerbated NAFLD in these mice via IFN-γ activity. Therefore, we crossed Prf^−/−^ mice with IFN-γ receptor-deficient mice to get double knockout mice (IFN-γR^−/−^ and Prf^−/−^). Following HFD challenge, IFN-γR^−/−^ and Prf^−/−^ mice gained similar amounts of body weight but had significantly decreased liver weights when compared to Prf^−/−^ mice (Figures 6A,B). Notably, IFN-γR^−/−^ and Prf^−/−^ mice showed significantly rescued NAFLD symptoms, including diminished hepatic steatosis, cellular ballooning, and lipid accumulation (Figure 6C). We also found that IFN-γR^−/−^& Prf^−/−^ mice had reduced serum ALT, cholesterol, and liver TG levels, as well as diminished pro-inflammatory cytokine production, while the level of IFN-γ was no significantly changed after IFN-γ receptor deficiency (Figure 6D). Moreover, the cell number of pro-inflammatory (F4/80^+^iNOS^+^) macrophage in the livers of IFN-γR^−/−^Prf^−/−^ mice was also dramatically decreased when compared to Prf^−/−^ mice (Figure 6E). Taken together, these findings strongly support an important role for elevated IFN-γ in promoting NAFLD progression in the context of perforin deficiency, given that ablation of IFN-γ signaling had a protective effect on the liver in an NAFLD mouse model.

### CD4 T Cells Demonstrate Intrinsically Elevated IFN-γ Production in Prf^-/-^ Mice

**Figure 7:**
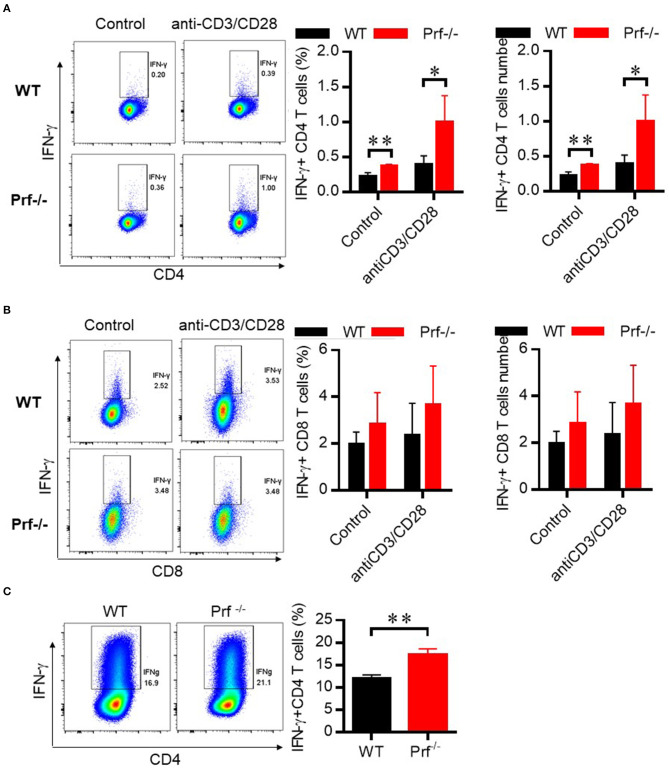
CD4 T cells in Prf^−/−^ mice have an intrinsically elevated ability of IFN-γ production. CD4 T cells **(A)** and CD8 T cells **(B)** from WT and Prf^−/−^ mouse spleens were cultured in media supplemented with GolgiStop in the presence or absence of plate-bound anti-CD3 plus anti-CD28 for 4–6 h and then stained for intracellular IFN-γ (left, percentage; right, cell number, n = 5–6). **(C)** Naive CD4+ T cells were sorted from the spleen of WT or KO mice and differentiated to Th1 cells for 3 days. The IFN-γ production in these cells was detected by flow cytometry. Representative FACS plots and total cell number of IFN-γ+CD4+T cells were shown. The data shown in each panel of the figures were collected from a single experiment, and each experiment was repeated for at least three times and rendered consistent results. Means ± SEM, *p < 0.05; **p < 0.01.

To define the functional properties of CD4 T cells from Prf^−/−^ mice, total spleen lymphocytes from WT and Prf^−/−^ mice were stimulated *in vitro* with anti CD3/anti-CD28 in the presence of Golgi-Stop. CD4 but not CD8 T cells from Prf^−/−^ mice showed increased levels of IFN-γ production upon CD3/CD28 stimulation (Figures 7A,B). To further study whether elevated IFN-γ production by CD4 T cells in Prf^−/−^ mice was an intrinsic property of these mice, naïve CD4 T cells were sorted from WT and Prf^−/−^ spleens and directly differentiated into Th1 cells. Interestingly, naïve CD4 T cells from Prf^−/−^ mice showed an elevated ability to differentiate into Th1 cells (Figure 7C). These findings support the conclusion that CD4 T cells undergo an intrinsic functional change in Prf^−/−^ mice.

## Discussion

HFD-induced NAFLD is a well-established mouse model for studying the pathophysiological mechanisms of human fatty liver disease. NAFLD is a prevalent liver disease worldwide that can have severe complications such as liver fibrosis and even development of hepatocellular carcinoma, for which there are no effective therapeutic approaches (28). Numerous factors such as leptin, TNF-α, and IL-6 are involved in the initiation and progression of hepatic steatosis and related metabolic dysfunction (29–31). However, the precise role of perforin, a cytotoxic factor released by T cells, has not been precisely studied in the context of HFD-induced NAFLD. Here we described an important protective role for perforin in regulating NAFLD progression. We found that perforin regulates intrinsic IFN-γ production in CD4 T cells, which influences pro-inflammatory macrophage accumulation to affect the progression of NAFLD.

One major finding of this study is the discovery of the protective role perforin plays in regulating NAFLD progression. Perforin is a ~67-kDa pore-forming protein that is stored in the secretory vesicles (granules) of CTLs and NK cells (32). Perforin is known to have potent and extensive functions in mediating targeted killing together with various other factors secreted by immune cells (19). Previous studies have shown that perforin-deficient mice are sensitive to obesity-induced insulin resistance as a result of restricted T cell expansion and activation in adipose tissue. Perforin has also been reported to play critical roles in promoting inflammation-mediated diseases, including type 1 diabetes (33), cerebral malaria (34), and viral myocarditis (35). A recent study revealed that perforin expressed in CD8 T cells regulates innate and adaptive immunity in the liver and exerts a protective effect in MCD (methionine/choline-deficient diet) diet-induced NASH models (36). Interestingly, MCD diet-induced non-obese NAFLD displays characteristics distinct from those of obesity-induced NAFLD. The precise role of perforin in liver metabolic disorders such as obesity-induced fatty liver disease has not been systematically researched yet. Using 14 month-old Prf^−/−^ mice fed either normal chow or HFD, we demonstrated that perforin played a critical protective role in obesity-induced NAFLD.

In our mouse experiments, we chose male mice fed on HFD (60% fat) for 10 weeks to induce NAFLD and found that Prf^−/−^ mice had more liver weight and liver TG accumulation in hepatocytes. These data are seemingly in contrast to a recent paper published by Cuff et al. which showed that after 24 weeks of obesogenic diet [22.6% fat, 23.0% protein, and 40.2% carbohydrate (w/w) supplement with sweetened condensed milk (Nestle) *ad libitum*], there was no difference in hepatomegaly and liver weight between the wild-type and perforin knockout female mice; otherwise, the fibrosis was significantly lower, and perforin KO mice suffer from less severe NAFLD mediated by NK cells (37). These conflicting findings may be due to the different diets, gender, and feeding time. In Cuff et al.'s paper, they chose 24 weeks as the timepoint so that they could compare the development of fibrosis, which is not usually pronounced at 10 weeks in NAFLD. Several reports indicated that free access to condensed milk induced an increase in serum AST activity and type I collagen deposition in the liver (38). NAFLD refers to a spectrum of liver diseases, including non-alcoholic fatty liver, which is characterized by steatosis with no or minor inflammation, and NASH, which is associated with inflammation and ballooning with or without fibrosis, and it may progress to liver cirrhosis and hepatocellular carcinoma (39, 40). The livers from mice fed a high-fat diet lacked fibrosis and showed mild steatosis and focal hepatocellular necrosis and apoptosis (41, 42). These contradictory findings suggest that perforin might have different actions at different stages during the pathogenesis of NAFLD and NASH.

Compared with WT controls, SFD-fed Prf^−/−^ mice showed increased liver TG levels at an early age (Figure 2C), which suggests that perforin may regulate early liver lipid accumulation independent of diet. After 10 weeks of HFD challenge, Prf^−/−^ mice had more IFN-γ-producing CD4 T cells in the liver. Further studies revealed that Prf^−/−^ mice had intrinsically increased IFN-γ-producing ability in CD4 T cells. However, it is still unclear how perforin, a cytotoxic factor that helps mediate target cell death, could stimulate CD4 T cells to produce IFN-γ. Further studies are needed to better understand this phenomenon.

The promotion of hepatic steatosis resulting from perforin deficiency was associated with a strong increase in hepatic macrophage accumulation and inflammation as evaluated by the expression of TNF-α, IL-6, and iNOS. Traditionally, macrophages are divided into pro-inflammatory (M1) and wound-healing (M2) classes. M1 macrophages, which are induced by IFN-γ and LPS and express pro-inflammatory cytokines such as TNF-α, IL-6, and IL-1β, are implicated in the pathogenesis of chronic liver inflammation. M2 macrophages, which are induced by IL-4, IL-10, and IL-13 and produce IL-10, TGF-β, PDGF, and EGF, have anti-inflammatory effects and promote wound healing (43–45). It is well established that macrophages play an important role during NAFLD pathogenesis. Previous studies found that depletion of macrophages with clodronate could significantly reverse NAFLD in mice (3, 46). Liver immune homeostasis is largely regulated within the hepatic sinusoid, where resident macrophages (Kupffer cells) are located as part of the liver reticuloendothelial system (also known as the mononuclear phagocyte system). This system forms a highly active, dynamic, and complex network, constituting the primary line of defense against invading microorganisms along with the involvement of other immune cells such as neutrophils. In different stages of liver disease, resident Kupffer cells and freshly recruited monocyte-derived macrophages play a key role in the regulation of inflammation, fibrogenesis, and fibrolysis (47). In our study, Prf^−/−^ mice had more macrophages, especially M1-type macrophage accumulation in liver after HFD challenge when compared with WT controls. However, we did not determine the mechanism of M1-type macrophage accumulation in the liver in this study. Is the increased accumulation due to the proliferation of resident Kupffer cells, or recruitment from peripheral circulatory systems, or the polarization of monocytes? We speculated that the increase in M1-type macrophage accumulation in the liver might be the result of monocyte polarization, since liver injury and lipid accumulation were almost non-existent in IFN-γR^−/−^ &Prf^−/−^ mice with decreased M1-type macrophage accumulation in the liver when compared with Prf^−/−^ mice. Further studies are necessary to better understand the mechanism behind this observation.

Depletion of CD4 T cells in Prf^−/−^ mice rendered these mice less sensitive to NAFLD, with similar levels of liver TG and macrophage accumulation detected when compared with WT controls. This finding highlights the indispensable role of CD4 T cells, especially Th1 cells, in NAFLD progression. In clinical studies, it was reported that the peripheral CD4 compartment in obese children displayed a Th1-prone phenotype, and pediatric patients with NASH also showed increased expression of IFN-γ in the liver. Dysregulated lipid metabolism in NAFLD was reported to cause a selective loss of intrahepatic CD4+ lymphocytes, leading to accelerated hepatocarcinogenesis (48). In this study, we demonstrated that the protective effect of perforin in HFD-induced NAFLD was almost completely dependent on Th1 cells, which is consistent with the existing literature.

In conclusion, we demonstrated that perforin acts as an important immune regulator in NAFLD progression through regulating INF-γ-producing CD4 T cells to decrease macrophage accumulation in the liver. Based on these findings, therapeutic strategies targeting perforin might be a promising approach for the development of novel strategies to prevent or treat hepatic steatosis and related metabolic disorders in the liver.

## Data Availability Statement

The datasets generated for this study are available on request to the corresponding author.

## Ethics Statement

The animal study was reviewed and approved by Laboratory Animal Ethics Committee Jinan University.

## Author Contributions

DL and QWa designed the project, performed experiments, and collected and analyzed the data. QWa wrote the manuscript. JZ and MZ worked on the mouse model. GC and JH helped modify and revise the article. QWe, HZ, and ZL maintained and genotyped the mice. LZ and QS performed RT-PCR. ZY, HY, and QWa supervised and coordinated the work, designed the overall research study, and helped write the manuscript. All authors have read, discussed, and approved the final manuscript.

## Conflict of Interest

The authors declare that the research was conducted in the absence of any commercial or financial relationships that could be construed as a potential conflict of interest.
